# Pediatric chronic non-bacterial osteomyelitis in Goteborg, Sweden

**DOI:** 10.1186/1546-0096-13-S1-P180

**Published:** 2015-09-28

**Authors:** S Berg, P Wekell, S Óskarsdóttir, J Martinell, R Rupröder, E Fridh, A Karlsson, T Backteman, A Fasth

**Affiliations:** 1 Göteborg University, Pediatrics, Göteborg, Sweden; 2 Pediatric Immunology and Rheumatology, The Queen Silvia Children's Hospital, Sweden, Sweden; 3 NU Hospital, Department of Pediatrics, Uddevalla, Sweden; 4 Södra Älvsborg Hospital, Department of Pediatrics, Borås, Sweden; 5 Pediatric Orthopedics, the Queen Silvia Children's Hospital, Göteborg, Sweden; 6 Göteborg University, Department of Rheumatology and Inflammation Research, Göteborg, Sweden

## Introduction

Chronic non-bacterial osteomyelitis (CNO) is today included among the autoinflammatory bone diseases. An alternative term in the literature is chronic recurrent multifocal osteomyelitis (CRMO). The etiology of the autoinflammatory bone diseases is unknown except for a few extremely rare monogenic diseases.

## Objectives

To describe a pediatric CNO cohort with respect to age at onset, age at diagnosis, number and location of lesions, imaging and treatment.

## Patients and methods

Patients with CNO treated at the Queen Silvia Children's Hospital, Goteborg from 2000 to 2015. It is a retrospective file review of patients diagnosed before the age of 18 years.

## Results

Twenty-seven patients with CNO were identified. The majority of the patients were females (22/27, 81%). The median age at onset of symptoms were 8.6 years (range 4.3 to 16 years) and the median age at diagnosis were 11.2 years (range 5.3 to 17.8). In total 80 lesions were found in the 27 patients. In 5 patients (19%) only one lesion was found. Most frequently lesions were found in proximal tibia (n=14), distal femur (n=11), clavicle (n=11), distal tibia (n=10) and vertebral column (n=5).

A biopsy was performed in 24 patients and it confirmed osteomyelitis in 22 (92%). A microorganism was identified in 6 cases, but was considered a non-significant finding and not the etiology of the osteomyelitis.

Plain x-rays were performed in all patients. In addition, 21 (78%) patients had a bone scan, 16 (59%) a MRT and 3 (11%) a whole body MRT.

Twelve patients (44%) were treated with antibiotics in an early phase of the disease.

Almost all patients were treated with NSAID (n=25, 93%) and many with a short course of corticosteroids (n=15, 59%). DMARDS were used in 14 patients including methotrexate that was used in 13 patients (48%) and sulfasalazine in 1 patient.

TNF inhibition was used in 6 patients and IL-1 blockade in 2 patients. Bisphosphonate was used in 3 patients.

## Conclusions

In our cohort of patients with CNO there is a predominance of females. The median age of onset is 8.6 years. There is a large diagnostic delay. About 1/5 of patients had only one lesion which, makes the term CRMO problematic. The severity of CNO ranges from only one mild lesion to a severe phenotype with many lesions. Some patients only needed treatment with NSAIDs while others required intense treatments with biologics or bisphosphonates.

